# Neural Network-Based Surrogate Modeling for Buckling Performance Optimization of Lightweight-Composite Collapsible Tubular Masts

**DOI:** 10.3390/biomimetics9080494

**Published:** 2024-08-14

**Authors:** Flavia Palmeri, Susanna Laurenzi

**Affiliations:** Department of Astronautical Electrical and Energy Engineering, Sapienza University of Rome, Via Salaria 851-881, 00138 Rome, Italy; flavia.palmeri@uniroma1.it

**Keywords:** lightweight structures, collapsible tubular mast (CTMs), buckling behavior, neural network (NN)-based surrogate models, multi-objective optimization (MOO)

## Abstract

The collapsible tubular mast (CTM) can be compactly folded for transport and deployed in orbit to serve as a key structural element. Once deployed, the CTM is vulnerable to buckling under axial load and bending moments, compromising its load-bearing capacity. The intricate relationship between the CTM’s cross-section and its buckling behavior poses a significant challenge for designers. This is due to the ultra-thin nature of the CTM, which gives rise to highly localized buckling modes rather than global ones. To overcome this challenge, we developed surrogate models using a neural network (NN) trained with data from finite element analysis (FEA). These NN-based surrogate models provide high computational accuracy in predicting nonlinear buckling loads under axial force and bending moments around the two principal axes of the CTM’s cross-section, achieving $R^{2}$ values of 0.9906, 0.9987, and 0.9628, respectively. These models also significantly improve computational efficiency, reducing prediction time to a fraction of a second compared to several minutes with FEA. Furthermore, the NN-based surrogate models enable the usage of the non-dominated sorting genetic algorithm (NSGA-II) for multi-objective optimization (MOO) of the CTMs. These models can be integrated in the NSGA-II algorithm to evaluate the objective function of existing and new individuals until a set of 1000 non-dominated solutions, i.e., cross-sectional configurations optimizing buckling performance, is identified. The proposed approach enables the design of ultra-thin CTMs with optimized stability and structural integrity by promoting design decisions based on the quantitative information provided by the NN-based surrogate models.

## 1. Introduction

The limited transport space and weight of the launch segment pose significant challenges on the integration of large-scale solar sails, deep space exploration antennas, and high-capacity solar panels for large satellites. To address these constraints, two main methods are employed: modular design strategies and deployable composite booms (DCBs). Modular design strategies involve creating systems with interchangeable modules, allowing for scalability by integrating additional modules to expand the system [1,2,3]. DCBs can be efficiently packed by coiling for transport in orbit [4,5,6,7,8], and, upon deployment, they serve as a fundamental structural component in various space systems such as parabolic mesh antennas [9], solar sails [10,11,12,13], roll-out solar arrays [14], probes [15], and docking systems [16]. In this work, we focus on DCBs. The DCB category encompasses a wide range of structures, which can be distinguished according to their cross-sectional shape into storable tubular extendible members (STEMs), triangular rollable and collapsible (TRAC) booms, sheath-based rollable lenticular-shaped and low-stiction (SHEARLESS) booms, and collapsible tube masts (CTMs). The STEM is a thin-walled composite shell with circular cross-section [17]. The TRAC boom is composed of two curved C-shaped section joined along one edge [18]. The SHEARLESS boom is formed by coupling the edges of two cylindrical shells front-to-front by a tightly fitted outer sheath [19]. The CTM is made by joining along the edges two omega-shaped shells. In this paper, we examine the CTM.

Given its closed cross-sectional shape, the CTM has garnered increasing attention from researchers since its inception. Initially proposed by B. Rennie for a metal alloy boom structure [20], the double-omega cross-sectional design transitioned about a decade later to carbon fiber reinforced polymers (CFRPs), marking the inception of CTMs under the European Space Agency (ESA) [21]. Since then, the CTM has attracted significant interest from major space agencies and corporations. The German Aerospace Center (DLR) considers the development of the CTM as a key technique for its solar sailing technology [22,23,24]. The National Aeronautics and Space Administration (NASA) has explored the potential of the CTM boom as a solar sail boom for low-cost deep space exploration [25]. DLR and NASA have collaborated in the Deployable Composite Booms project, focusing on refining design methods and manufacturing techniques for CTMs, as well as developing mechanical systems necessary for their storage and deployment [26]. The DCB technology developed will be tested through NASA’s Advanced Composite Solar Sail System (ACS3) project, which is currently ongoing [27].

Thanks to the research effort devoted to the study of CTMs, various key aspects of their behavior have been understood through the years. A particular emphasis has been placed on their response to various mechanical loads, with a focus on those encountered during packaging and operations.

During packaging, the cross-section of the CTMs must be flattened, and then the CTMs must be coiled along their length. Excessive stresses may arise during these critical processes, potentially causing damage to the CTM material. To have a more grounded understanding of these processes, Hu et al. performed an in-depth analysis using experimental, numerical, and analytical techniques to investigate the mechanical response of CTMs under both compressive and tensile flattening deformations [28]. Chen et al. conducted experiments to study the behaviors of large deformations during flattening and wrapping processes [8]. They developed three-dimensional finite element method (FEM) models that accurately predicted the mechanical characteristics observed in their experimental investigations. Bai et al. established analytical models to predict folding moment of CTM, validating their analytical results against experimental findings [29]. Additionally, they established an analytical model for the arising in-plane strain and interlaminar shear stress [30]. Similar investigations were conducted by Liu et al., who numerically and experimentally investigated the flattening and coiling processes, as well as stress distribution, of a shape memory composite CTMs [31]. To prevent composite material failure during critical folding and coiling processes, Palmeri et al. introduced a novel interface design for CTMs [32]. Their approach involves strategically removing material from the fixed end of the CTM to mitigate high localized curvature that may arise during packaging.

During operations, CTMs serve as supporting structure for large-scale space systems. Particularly, CTMs guide these systems in retrieving their operational configuration by deployment, and subsequently serve to maintain this configuration. Once deployed, CTMs are subjected to axial load and bending moments, which can lead them to buckle. Many recent studies have been conducted on this topic, with some focusing on buckling behavior under pure axial loading [33,34], and others with emphasis on buckling behavior under pure bending [35]. Hu et al. performed linear and non-linear buckling analysis of CTMs under axial loading, comparing numerical findings with experimental results [33]. The estimated critical buckling load from finite element analysis (FEA) was found to be considerably higher than experimental measurements. Introducing initial imperfections based on experimental observations and performed post-buckling analyses, resulted in a closer alignment with experimental findings. Yao et al. performed a correlation analysis between numerical model and analytical prediction for CTM subjected to axial loading [34]. Based on Euler’s formula and laminated theory, they derived the critical buckling load formula of CTMs. The correctness of this formula is verified against FEA, showing its applicability only to the CTMs with a slenderness ratio greater than 64. Jia et al. presented an automatic FEA to perform simulation over 50 cross-section configurations scheme, accounting for high geometric nonlinearity [35]. Finally, they constructed the design space against non-linear buckling under pure bending moments.

Although multiple authors have investigated the CTM buckling behavior, there remains a lack of reliable predictions for the buckling loads, especially in relation to variations in cross-sectional configuration. In the context of buckling under axial loading, the influence of the cross-sectional configuration was analyzed treating the cross-sectional parameters as continuous variables. However, this approach was taken only for very slender CTMs. In fact, Yao et al. presented a parametric study using an analytic-based approach, but the derived buckling load formula was valid only for CTMs with a minimum slenderness ratio of 64 [34]. In the context of buckling under bending moments, the influence of the cross-sectional configuration was investigated but accounted only for discrete cross-sectional parameters values. Jia et al. carried out the largest computational investigation of CTM buckling under bending moments, performing systematically simulation over 50 different cross-sectional configurations [35]. However, to the best of our knowledge, no investigation of the buckling behavior under bending has considered cross-sectional parameters as continuous variables. If predictions of buckling loads were available, the buckling performance of the next generation of CTMs could be optimized during the design and construction phases. Therefore, in this study, we aim to bridge this gap by developing surrogate models of the buckling behavior of CTM under both axial loads and bending moments, with particular emphasis on variations in cross-sectional configurations. Once those models are available, a multi-objective optimization (MOO) can be performed, to find the cross-sectional configuration that optimizes the buckling performance of CTMs under different loading conditions.

Currently, various surrogate models have been developed to explore a wide range of DCBs under different loading conditions. These models have been used to optimize the designs. Yang et al. developed surrogate models for deployment stiffness and folding behavior, investigating designs such as tubular DCBs, TRAC booms, N-shaped DCBs, and CTMs [36,37,38,39]. They conducted FEA on different designs and established surrogate models, on the basis of FEA results, using Response Surface Methodology (RSM) and a neural network (NN). Optimization of the designs was carried out using a genetic algorithm (GA). Similarly, Shi et al. optimized the M-shaped DCB using a combined RSM and GA technique [40]. The RSM method was used to fit the FEA data to obtain surrogate models for the strain storage and the fundamental frequency in the deployed state. Those models are fed into a GA for MOO, aiming at maximizing the strain stored energy and the fundamental frequency. Zhang et al. established a model of the initial snap-through and coiling process of a tubular DCB using RSM, and subsequently employed a GA to optimize the mechanical responses of the structure [41]. Bessa and Pellegrino proposed a surrogate model to predict the imperfection-sensitive buckling moments obtained when bending the TRAC booms. They combined Bayesian regression and uncertainty quantification to predict the mean value and standard deviation of the quantity of interests, which are then used in MOO to guide TRAC boom design [42]. A few surrogate models have been developed based on neural networks (NNs) to optimize the design of deployable composite hinges (DCHs) [43,44]. Liu et al. minimized the peak folding moment and mass while maximizing the peak torsional moment by using RSM and machine learning techniques, along with state-of-the-art GAs to obtain optimal DCH designs. Jin et al. maximized both the maximum stored strain energy during folding and the maximum bending moment during deployment of the DCH using data-driven surrogate modeling and shape optimization. Although NN-based surrogate models have demonstrated significant potential for DCHs, research on their application to DCBs remains elusive, and their use for CTMs modeling is still unexplored.

As in the above-mentioned works [36,37,38,39,40,41,42,43,44], to built the surrogate models, we gather data on the structure responses under various input conditions via FEA. Particularly, we generate input design samples, and run three FEM simulations per each design sample to determine the non-linear buckling load under three loading scenarios: axial loading and pure bending around the two cross-sectional principal axis. The data collected is thus used to train a neural network (NN), with the final aim of providing NN-based surrogate models. The NN-based surrogate models are then embedded into a MOO process. Optimal cross-sectional geometries are identified using the bio-inspired algorithm known as the non-dominated sorting genetic algorithm (NSGA-II).

## 2. Materials and Methods

**Figure 1:**
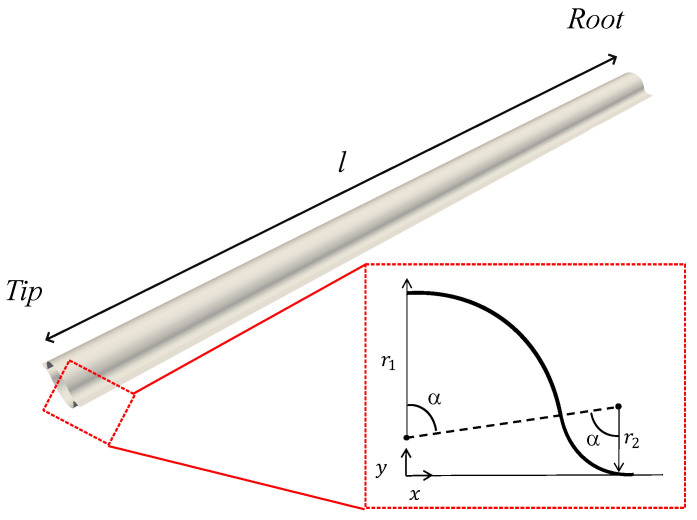
Geometric description of the CTM: $r_{1}$ is the radius of the central arc-segment, $r_{2}$ is the radius of the edge arc-segment, α is the subtended angle of both the central and edge arc-segment.

The CTM under investigation is represented in Figure 1 in its deployed configuration. It is made by two 500 mm long bi-convex thin shells, bonded along their edges. The cross-section of each shell is composed of three arc segments and two flat segments in correspondence of the bonding area. The central arc segment is associated with the radius of curvature, $r_{1}$, and the subtended angle, $\alpha_{1}$; the edge arc segment is associated with the radius of curvature, $r_{2}$, and the subtended angle $\alpha_{2}$; the flat segment is associated with its length, *w*. The subtended angles of both arc segments must be equal ($\alpha_{1}=\alpha_{2}=\alpha$), to prevent kinks and maintain tangent continuity.

The CTM cross-section in its flattened state is fully described by the length of the shell *h*:

(1) $$h=2(L_{1}+L_{2}+w)$$

where:

(2) $$L_{1}=r_{1}\alpha$$

(3) $$L_{2}=r_{2}\alpha$$

To ensure a fair comparison between different cross-sectional configurations, we consider the same flattened height, h=130 mm, for every design. Furthermore, the bonding region width is kept constant, w=4.5 mm. Since *h* and *w* are known quantities, and Equation (1) must be satisfied, only two of the three geometric parameters $r_{1}$, $r_{2}$, and α can be chosen as design variable. Particularly, we allowed $r_{1}$ and α to freely vary, and then we computed $r_{2}$ accordingly to the following:

(4) $$r_{2}=\left(h-2w-2r_{1}\alpha \right)/\left(2\alpha \right)$$

In accordance with previously reported data for TRAC booms [45], each CTM omega shell consists of a laminate with $\left[45_{GF_{PW}}/0_{CF}/45_{GF_{PW}}\right]$ stacking sequence, where $GF_{PW}$ denotes the JPS E-glass fabric plain weave prepreg with Patz PMT-F4 epoxy resin, while CF represents a unidirectional Torayca T800 carbon fiber prepreg tape with NTPT ThinPreg 120 EPHTg-402 epoxy resin. The thickness of each CTM omega shell laminate is about 80μm. The bonding region is composed of a laminate with the stacking sequence $\left[45_{GF_{PW}}/0_{CF}/{\left(45_{GF_{PW}}\right)}_{3}/0_{CF}/45_{GF_{PW}}\right]$, achieved by bonding two CTM shells with a $GF_{PW}$ lamina oriented at 45°.

The material properties sourced from [45]. Particularly, the stiffness properties of the laminate of the CTM shells are modeled according the *A* and *D* matrix collected below:

(5) $$A=\left(\begin{array}{ccc} 5432 & 619 & 0\\ 619 & 942 & 0\\ 0 & 0 & 737 \end{array}\right)\;N/\mathrm{mm}$$

(6) $$D=\left(\begin{array}{ccc} 1.076 & 0.482 & 0\\ 0.482 & 0.781 & 0\\ 0 & 0 & 0.459 \end{array}\right)\;\mathrm{Nmm}$$

and the corresponding matrices for the bonding region, denoted by $A_{w}$ and $D_{w}$, are given as follows:

(7) $$A_{w}=\left(\begin{array}{ccc} 11369 & 1512 & 0\\ 1512 & 2269 & 0\\ 0 & 0 & 1727 \end{array}\right)\;N/\mathrm{mm}$$

(8) $$D_{w}=\left(\begin{array}{ccc} 28.20 & 4.32 & 0\\ 4.32 & 7.44 & 0\\ 0 & 0 & 4.93 \end{array}\right)\;\mathrm{Nmm}$$

**Figure 2:**
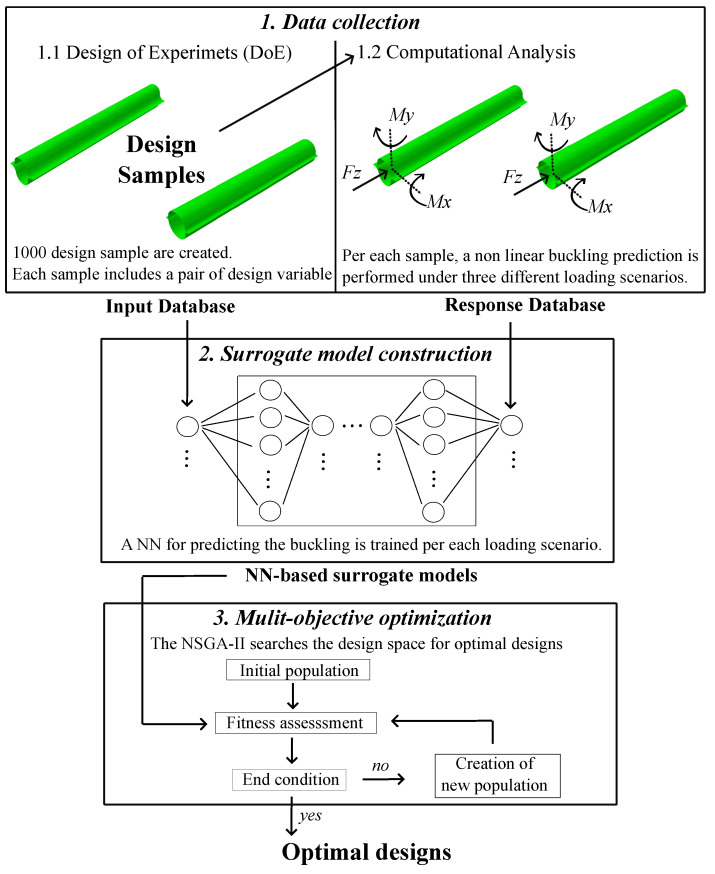
Workflow of the proposed methodology consisting of three steps: data collection, surrogate model construction, and multi-objective optimization.

In this study, we focus on finding the combination of cross-sectional parameters $r_{1}$ and α that maximize the nonlinear buckling loads of the CTM considering idealized geometries, i.e., without imperfections. To this extent, the proposed methodology consists of three steps: data collection, surrogate model construction, and MOO. The data collection steps consists of two stages: design of experiment (DoE) and creation of the response database. Thorough the DoE process, a set of initial design samples is created and the corresponding response database is then evaluated through computational analysis via FEA. The design samples and response database constitutes the data collection which serves as the basis for constructing the surrogate models. The construction of the surrogate models consists of building models capable of accurate prediction for the buckling behavior. The NN-based surrogate models are built by training a NN with the data acquired during the data collection step. The predictive capability of the NN-based surrogate models are validated using statistical error function. Finally, a MOO is performed to find optimal cross-sectional designs that maximize the buckling loads of the structure. For the MOO process, the NSGA-II algorithm is used. Each individual in the NSGA-II consists of a set of the variables fully describing the cross-sectional geometry. The surrogate models are embedded into the NSGA-II algorithm to assess the buckling loads value of each individual, which serves as their fitness score. Iteration after iteration new individuals are generated through selection, cross-over and mutation mechanisms, and these individuals are evaluated using the surrogate models to determine their objective values. The iteration stops when the optimal designs are found. The workflow of the proposed methodology is shown in Figure 2.

A similar workflow, encompassing design of experiments where sampling designs are created, computational analyses of each design sample where quantities of interest are investigated using FEA, surrogate model construction, and MOO, has been followed in previous research [36,37,38,39,40,41,42,43,44]. Most of earlier works employed RSM to build surrogate models [36,37,38,40,41]. Yang et al. developed surrogate models for coiling and deploying tubular booms through RSM [36]. Using the same methodology, they also created surrogate models for the wrapping peak moment, maximum stress, and fully deploying fundamental frequency for a TRAC boom, as well as for bending stiffness around the *x*- and *y*-axes and torsional stiffness around the *z*-axis for an N-shaped boom [37,38]. Similarly, Shi et al. developed surrogate models for strain storage and fundamental frequency in the deployed state of an M-shaped boom [40], while Zhang et al. modeled the initial snap-through and coiling processes of a tubular DCB [41]. In contrast, Bessa and Pellegrino used Gaussian process regressions (GPR) to build surrogate models for the buckling behavior of TRAC [42]. To date, only Hui Yang et al. have employed NN-based surrogate models for DCBs [39]. Specifically, they developed surrogate models for the coiling peak moment and maximum principal stress in a four-cell lenticular honeycomb deployable (FLHD) boom. However, NN-based surrogate models have been used by Liu et al. and Jin et al. to optimize DCHs [43,44]. All the aforementioned studies culminated in an optimization design process, with most utilizing the NSGA-II algorithm [37,38,39,40,41]. The sequential quadratic programming algorithm was also used for optimization purposes in [36]. Some of these studies performed benchmarks between different optimization algorithms [42,43,44].

The subsequent subsections describe, in detail, each step of the proposed methodology applied to the optimal design of CTM cross-section.

### 2.1. Data Collection

In this subsection, the data collection step is described, detailing each of the two stages involved separately. Following the order in which the stages are performed, the Design of Experiments (DoE) process is presented first, followed by the computational analysis phase.

In the DoE, sample designs are created by sampling the design space. In this work, the design space is made of two design variables: the radius of the central arc segment, $r_{1}$, and the subtended angle of both arc segments, α. The radius $r_{1}$ has a lower limit due to the high strain that may occur during folding and a upper limit determined by the length of the flattened shell being h=130 mm. The angle α has a maximum allowable value for manufacturing reasons. Those limits have been indicated in [46]. A minimum value for the angle α has also been set. The design variables, alongside with their lower and upper bounds, are collected in Table 1.

Once the design space is identified, various methodologies are available to perform sampling. The Sobol sequence and Latin Hypercube sampling are commonly used approaches [47,48]. In alignment with prior research, the Sobol sequence has been chosen for the DoE process in this study [42]. The Sobol sequence generates sample designs that are evenly distributed, minimizing clustering and avoiding gaps effectively. Each sample design is characterized by a pair of design variables, i.e., $r_{1}$ and α. Knowing $r_{1}$ and α values, $r_{2}$ is computed according to Equation (4). The same bonds identified for $r_{1}$ must be enforced for $r_{2}$. If the $r_{2}$ value falls between the limits depicted for $r_{1}$, the sample design is kept. Otherwise, it is discarded.

At the end of the DoE process, 1000 sample designs are created.

Following the DoE, the next phase of data collection consists of the computational analysis of each DoE point. The computational analysis involves FEM simulations conducted using the commercial software Abaqus 2020, which is widely utilized for predicting local buckling in a variety of structures [49,50,51,52,53].

For each design sample, three models are developed to study the buckling behavior under different loading conditions: axial load and bending moment around the two principal axis of the CTM cross-section.

In every model, the CTM is modeled by sketching its cross-section geometry in the x−y plane and then by extruding it to the desired length of 500 mm along the *z* direction. The elements used for the CTM are S4R: four-node shell elements with reduced integration (and hourglass control). The material properties for the CTM shell are given through the stiffness matrices in Equations (5) and (6), while the material properties for the bonding region are given through the stiffness matrices in Equations (7) and (8). A material-oriented coordinate system, aligned with the longitudinal and transverse coordinates of the CTM, is established for the shell elements.

**Figure 3:**
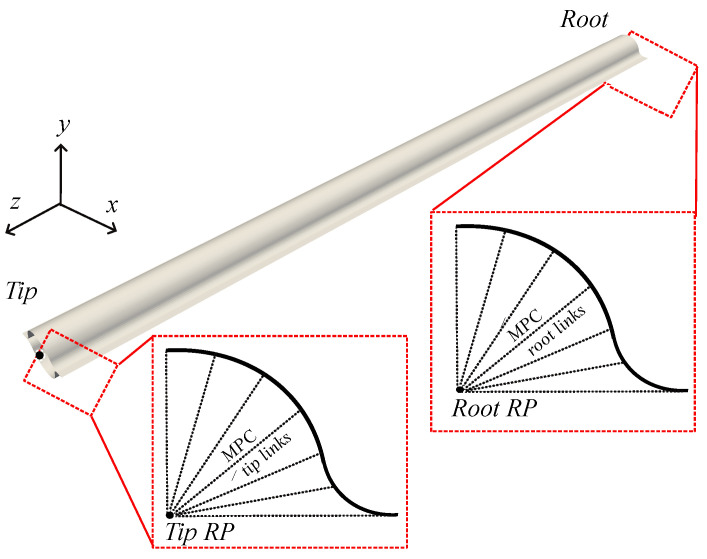
Multi-Point Constraints (MPCs) used in the present study to apply boundary conditions (BCs). The edge nodes at the root cross-section and the edge nodes at the tip cross-section have been associated with a root and a tip reference point (RP), respectively.

For every model, the boundary conditions (BCs) vary accordingly to the loading condition. To easily vary the BCs from one model to another, we use Multi-Point Constraints (MPCs) to define BCs. The MPC links the motion of follower points to the motion of a single reference point (RP). The edge nodes at the root cross-section and the edge nodes at the tip cross-section have been associated with two RPs, namely the root RP and the tip RP. The MPCs with the corresponding RPs are shown in Figure 3.

For all the three models, the RP to which the nodes on the root edge are linked is constrained in x,y,z translation and x,y,z rotation to simulate the interlocking of the boom with the spacecraft. At the other RP, the BCs applied vary for each model, depending on the loading condition. In the scenario of axial loading, a unitary axial force along the *z*-axis, $F_{z}$=, is applied to the tip RP, and only the rotational degree of freedom (DoF) around *z* is fixed. In the scenario of a bending moment around the *x*-axis, a unitary bending moment around the *x*-axis, $M_{x}$, is applied to the tip RP, the transitional DoF along *x* and the rotational DoF around *y* and *z* are fixed, while the others are left free. In the case of a bending moment around the *y*-axis, a unitary bending moment around the *y*-axis, $M_{y}$, is applied to the tip RP, the transitional DoF along *y* and the rotational DoF around *x* and *z* are fixed, while the others are left free.

For all models, a well-established approach from the literature is used to predict the non-linear buckling load of the CTM under the various loading scenarios [35,42,45]. The approach consists of performing three analyses subsequently: firstly, a linear buckling analysis is performed, then a non-linear static analysis is carried out and, finally, a sequential linear buckling analysis concludes the procedure. In the first linear buckling analysis, a linear bifurcation analysis is performed on the undeformed configuration of the CTM to provide an initial prediction for the buckling load. Subsequently, a nonlinear static analysis is performed under load control using a large displacement formulation to capture geometric non-linearity. The linear buckling load given as output of the precedent linear buckling analysis is applied to the CTM, and the simulation proceeds without stabilization up to that load or until convergence failure due to buckling occurs. Finally, a sequential linear buckling analysis is conducted, which is a linear bifurcation analysis performed on the deformed CTM. The deformed CTM shape is attained from the non-linear static analysis, starting from the gradually decreasing last available increment until reaching an increment where the buckling load can be extracted, i.e., an increment where the structure is close to but not in the post-buckling regime. This approach guarantees that linear perturbation analysis effectively determines buckling mode shapes and loads/moments near the buckling point, accurately predicting the buckling behavior of even nonlinear structures [42].

The procedure for non-linear buckling estimation has been implemented into a Python script within Abaqus software. This Python script automates the simulation process, enabling the handling of simulations consisting of three sequential analyses, over 1000 sample designs, and under three different loading scenario. The results of these simulations, which are of interest, are saved in a database that is subsequently used for constructing the surrogate models.

### 2.2. Surrogate Model

Each surrogate model is implemented using a NN approach [54]. In this work, we use a fully connected NN (FCNN) with a structure consisting of multiple layers: the input layer, several hidden layers, and the output layer. Each layer in a FCNN includes multiple neurons, with each neuron connected to all neurons in the preceding and subsequent layers. These connections have associated weights, which determine the influence of one neuron on another. The neuron calculates a weighted sum of its inputs and applies an activation function to produce an output. The output of the last layer, which is the output layer, represents the prediction of the NN based on the initial input data, which is compared to the actual value to obtain an error. During training, weights are adjusted through algorithms to minimize the error in the NN’s output, enabling the NN to learn and make accurate predictions.

The quality of NN can be assessed through evaluation indices such as: the mean square error (MSE), which measures the deviation between the predicted value and the true value; the mean absolute error (MAE), which represents the discrepancy between the true and predicted values; and the R-squared ($R^{2}$) value, which reflects how well the predicted values match the true values, with a value closer to 1 indicating a better fit. The MSE, MAE, and $R^{2}$ are used herein to check if the NN-based surrogate models constructed are capable of accurate predictions.

The construction of the NN-based surrogate model begins with selecting appropriate NN hyper-parameters such as: the initialization of weights and the algorithm for weights updates, the activation function, the regularization technique, and the NN architecture. These hyper-parameters have great effects on the accuracy of the NN-based surrogate model and should be optimized to improve performance. The optimization of such hyper-parameters involves an iterative trial-and-error process, aimed to balance complexity and efficiency.

In the present work, the weights of each layer are initialized using the He uniform initialization [55]. According to the He uniform initialization, weights are drawn from a uniform distribution:

(9) $$W∼U\left(-\sqrt{\frac{6}{n_{in}}},\sqrt{\frac{6}{n_{in}}}\right)$$

where $n_{in}$ is the number of neurons in the preceding layer. Those weights are updated according to the Adaptive Moment Estimation (Adam) algorithm [56].

For the current work, after each layer, the Rectified Linear Unit (ReLU) activation function is performed [57]. The ReLU activation functions can introduce non-linearity into the model, enabling the NN to learn and represent complex relationships between inputs and outputs. The ReLU function is defined as:

(10) $$\mathrm{ReLU}\left(x\right)=\left\{\begin{array}{cc} x & \mathrm{if}\;x\geq 0\\ 0 & \mathrm{if}\;x<0 \end{array}\right.$$

The regularization techniques are used to prevent overfitting, where the NN becomes too specialized in the training data and performs poorly on new data. In this work, dropout is applied to randomly disable a fraction of the neurons during training, preventing them from relying too much on each other and thus improving the model’s robustness [58].

We have chosen the He uniform distribution for weight initialization, the Adam algorithm for weight updates, the ReLU as the activation function, and the dropout as the regularization technique, as these hyper-parameters are commonly used in neural networks. Indeed, various examples of their usage are available in the literature across a wide range of domains, such as remote sensing [59,60], structural surrogate modeling [43,44], biomedical imaging [61], climate change modeling [62], and natural language processing [63].

**Figure 4:**
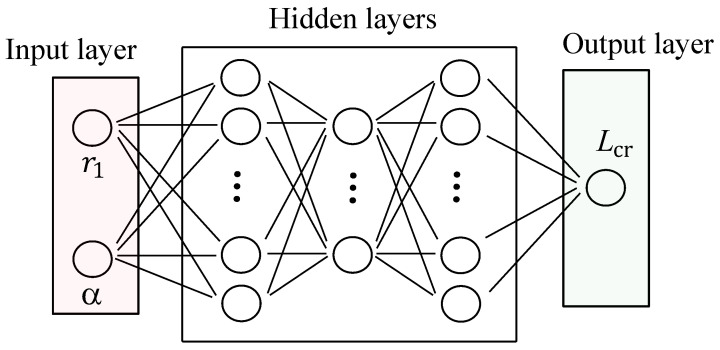
Neural network (NN) architecture including the input layer, the hidden layers, and the output layer. The neurons in the input correspond to the design variables, which are the radius of the central arc segment, $r_{1}$, and the subtended angle of both arc segments, α. The output layer is composed of a single neuron representing the predicted non-linear buckling load.

Finally, determination of the architecture of the NN includes determining the input layer, the output layer, and the hidden layers, and the corresponding number of neurons for each layer. The number of neurons in the input and output layers can be determined according to the number of input variables and output variables. In this paper, the input variables are the radius of the central arc segment, $r_{1}$, and the subtended angle of both arc segments, α, while the output variable is the non-linear buckling prediction. A generic architecture with the specific input and output layer for the present study is shown in Figure 4.

The number of hidden layers and of the corresponding neurons, used in the NN-based surrogate models, has been assessed through a fine-tuning process. Particularly, to select the NN architecture, 16 architectures are investigated and compared. These architectures can be distinguished into four different categories based on the number of layers (increasing from 1 to 4) and four different categories based on the total number of neurons (8-16-32-64). Each NN architecture has been designed with a central symmetry criterion, streamlining the trial-and-error process and enhancing the efficiency of NN design and implementation. The results of this comparison and the subsequent NN architecture of choice will be presented later in this paper.

Each NN was trained using the MEA loss function, with a maximum of 1000 epochs and an automatic early stopping criterion based on a patience parameter of 200 epochs.

The NN-based surrogate model has been trained using the previously collected database, with 80% of the data allocated for training, of which 85% was used for training, 15% for validation, and the remaining 20% for testing. These datasets were normalized to a range of [0, 1] as follows:

(11) $$x_{\mathrm{norm}}=\frac{x-x_{\min}}{x_{\max}-x_{\min}}$$

where $x_{\mathrm{norm}},x,x_{\min},$ and $x_{\max}$ denote the normalized value, the original value, the minimum value, and the maximum value of the data, respectively.

The NN-based surrogate models have been implemented in Python using TensorFlow [64].

### 2.3. MultiObjective Optimization

The NN-based surrogate models are then used to perform a MOO, with the aim of identifying a range of trade-off solutions to achieve global optimization of buckling performance. The set of trade-off solutions is best known as the Pareto non-inferior solution set, and its graphical representation in the objective space is referred to as the Pareto front. A MOO problem can be described as follows:

Minimize(12)f1(x),f2(x),…,fm(x)Subject to(13)  gj(x)≤0,∀j=1,2,…,p(14) hk(x)=0,∀k=1,2,…,q(15)    xmin≤xi≤xmax,∀i=1,2,…,n

where $f_{1}\left(x\right),f_{2}\left(x\right),\ldots ,f_{m}\left(x\right)$ denote the objective functions, $g_{i}\left(x\right),h_{k}\left(x\right)$ are the inequality and equality constraints, respectively, and x represents the decision variable vector. The primary goal is to minimize each objective function $f_{i}\left(x\right)$ over the feasible solution space. The decision variable vector x comprises design variables. These variables are subject to certain limitations. Particularly, $x_{\min}$ and $x_{\max}$ are their lower and upper bounds, respectively. The inequality and equality constraints require specific combinations of design variables to comply with established relationships, thus imposing additional restrictions on the feasible solution space.

In this study, we established a total of three objective functions:

- Maximize the non-linear critical buckling load under pure axial loading.
  (16) $$f_{1}\left(x\right)=-F_{cr}$$
- Maximize the non-linear critical buckling moment under pure bending about the *x*-axis.
  (17) $$f_{2}\left(x\right)=-M_{xcr}$$
- Maximize the non-linear critical buckling moment under pure bending about the *y*-axis.
  (18) $$f_{3}\left(x\right)=-M_{ycr}$$

Note that maximizing a function f(x) is equivalent to minimizing −f(x).

The design space is the same depicted in Section 2.1, with the design variable being the radius of the central arc, $r_{1}$, and the subtended angle of both arc segments, α. The design variables are treated as continuous variables within the upper and lower bounds, previously detailed in Table 1.

The feasible solution design space is further restricted by a total of two inequality constraints:

- $r_{2}$ has a lower bound
  (19) $$r_{2}>12\;\mathrm{mm}$$
- $r_{2}$ has an upper bound
  (20) $$r_{2}<50\;\mathrm{mm}$$

to ensure that the radius of the edge arc segment $r_{2}$ is also within the acceptable values according to the previous literature.

The MOO was carried out using pymoo, a high-performance Python-coded algorithm toolbox [65]. Among the algorithms available in the toolbox, we chose an elistic non-dominated sorting genetic algorithm, best known as NSGA-II [66]. We have chosen to employ the NSGA-II algorithm due to its extensive application in the optimization of DCBs [37,38,39,40,41]. Furthermore, a recent study has demonstrated the effectiveness of the NSGA-II algorithm in optimizing the design of CTMs [67]. Specifically, Liu et al. used the NSGA-II to minimize the ultimate coiling radius, the folding moment, and the linear density of a CTM, while maximizing the moment of inertia about the principal axes of its cross-section. The NSGA-II algorithm achieved superior performance compared to four other state-of-the-art algorithms evaluated during the optimization process. The objective functions are evaluated through the NN-based surrogate models, which are fed into the NSGA-II algorithm.

The NSGA-II uses real-valued coding. Initial points are sampled using the LHS, which divides the input space into cells and randomly selects one point in each cell along each dimension. Parents are then chosen using the binary tournament selection operator. It randomly selects two individuals from the population, and chooses the one demonstrating superiority across multiple objectives for reproduction. Once parents are selected, the crossover operation is implemented by using the simulated binary crossover (SBX) algorithm [68]. The SBX operator simulates the single-point crossover operator on binary strings to create two offspring solutions. This operator uses a distribution index $\eta_{c}$; higher $\eta_{c}$ values produce offspring closer to the parents, and lower $\eta_{c}$ values produce more diverse offspring. For the present study, we use a distribution index $\eta_{c}$ of 15 for the crossover, as it is the default value in the pymoo toolbox for the SBX operator when using NSGA-II. The NGSA-II automatically removes duplicates from offspring populations, repeating the mating process until the desired number of unique offspring is achieved. Finally, the polynomial mutation (PM) operator is applied to introduce variation in the generated solution thus ensuring population diversity [68]. The PM operator follows the same distribution as the SBX, and is characterized by a probability of execution. For this study, we use a distribution index $\eta_{c}$ of 20 for the mutation operator, as it is the default value in the pymoo toolbox for the PM operator when using NSGA-II. Additionally, the probability of applying the PM operator, referred to as the mutation rate, is set to 0.9, which is also the default value. Iteration after iteration, new individuals are generated through binary tournament selection, simulated binary cross-over and polynomial mutation, until a total number of 100 generations has been reached.

To find the optimal population size for the NSGA-II algorithm, we evaluated population of 250, 500, 750, and 1000 individuals. As the population size increases, more design points appears on the Pareto front. Therefore, we select the population size to be 1000. The number of offspring is set equal to 100. All the hyper-parameters are collected in Table 2.

For constraint handling, we use the feasibility-first approach [69], which prioritizes feasible solutions by comparing infeasible ones based only on their constraint violation values.

The MOO was carried out on a personal computer equipped with 16 GB of RAM, an AMD Ryzen 7 5800H with Radeon Graphics 3.20 GHz CPU, and an NVIDIA GeForce RTX 3060 Laptop GPU with 6 GB of dedicated RAM.

## 3. Results

In this section, the results of the combined NN-based surrogate models and MOO methodology are presented. Firstly, the design samples obtained through the DoE process and the corresponding response database evaluated through computational analysis are reported and discussed. Then, the NN-based surrogate models are presented, and their predictive capabilities are assessed through statistical error function. Finally, the designs for global optimization of the CTM buckling performance, which have been identified by feeding the NN-based surrogate models into the NSGA-II algorithm, are commented.

### 3.1. Data Collection Results

In this subsection, the results of the data collection are reported. The results of the DoE process and computational analysis are presented, following the order in which the two stages are performed.

**Figure 5:**
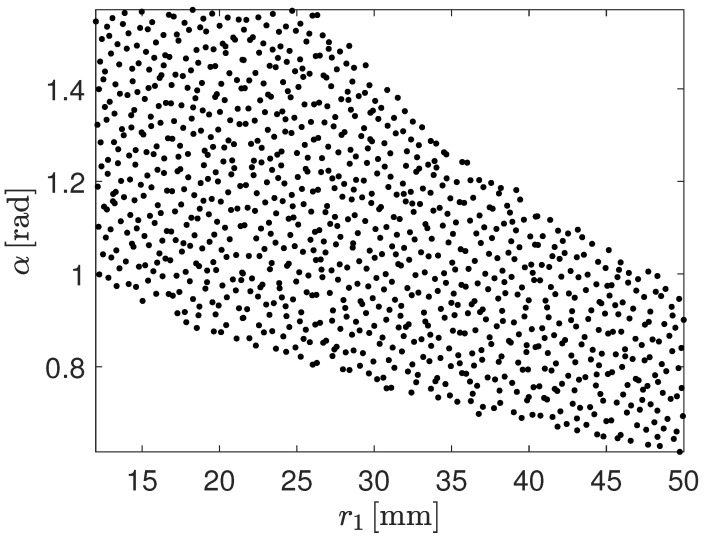
Design samples, in terms of the design variables $r_{1}$ and α, to determine the influence of the cross-sectional configuration on the non-linear buckling behavior of the CTM. The design samples has been built as a Sobol sequence.

Figure 5 displays the 1000 DoE points generated using the Sobol sequence applied to the two design variables, $r_{1}$ and α. The design samples do not span the entire design space that would be covered if solely the constraints on $r_{1}$ and α were considered. In fact, the lower and upper limits for $r_{1}$ must also be enforced for $r_{2}$. Given the need to validate the $r_{1}$ and α pairs to ensure that the corresponding $r_{2}$ value falls within the limits for $r_{1}$, the design space is further restricted beyond the constraints on $r_{1}$ and α alone, as shown in Figure 5.

The three simulation models developed to study the buckling behavior of each sample design are executed, and the response of each sample design subjected to axial load, bending moment around the *x*-axis, and bending moment around the *y*-axis has been collected and analyzed.

**Figure 6:**
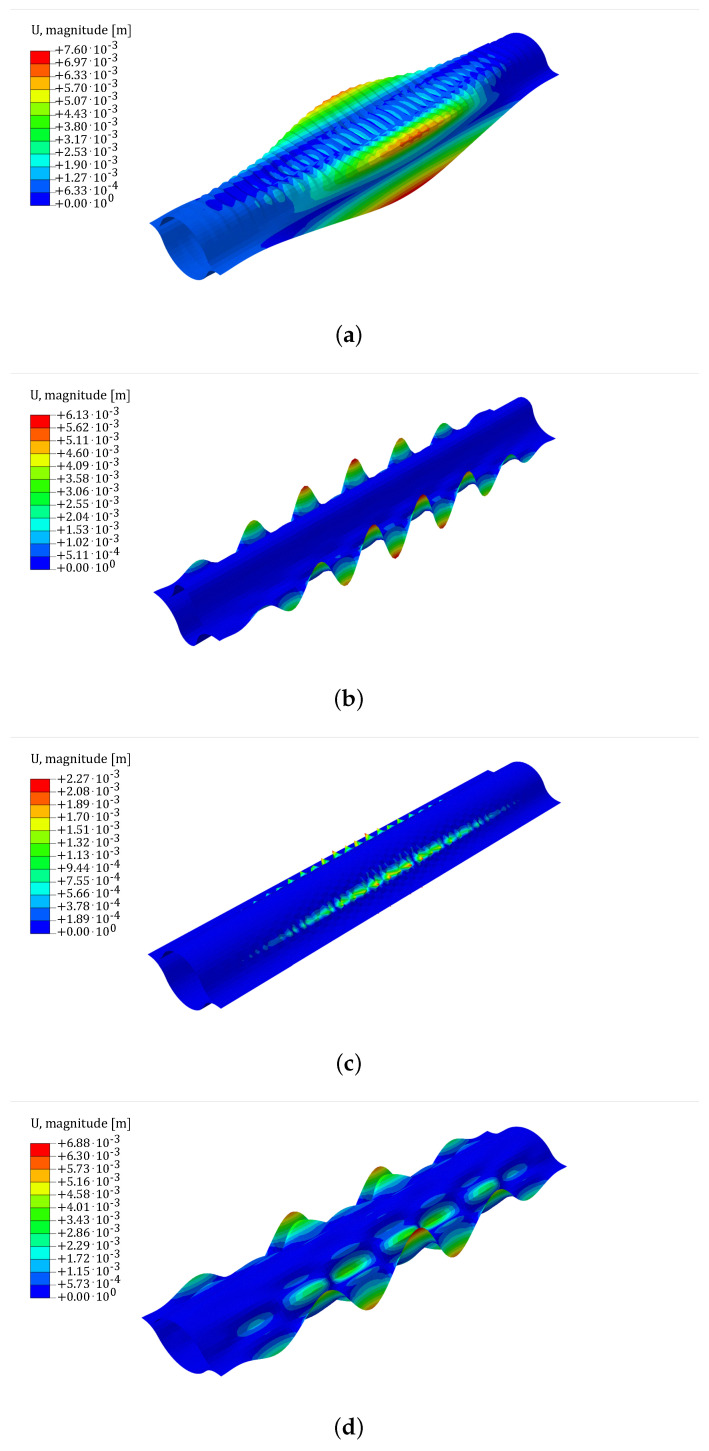
Buckling patterns for non-linear buckling in the axial loading scenario: (**a**) Wrinkles on central arc segment. (**b**) Wrinkles on edge arc segment. (**c**) Wrinkles on web region. (**d**) Wrinkles on a wide region including edge arc segment and web region. The scale-plate is shown in the figure with the magnitude of displacements expressed in m. In the deformed configuration, the magnitude of displacements has been amplified by a factor of 5.

For the scenario where an axial force is applied, four distinct non-linear buckling patterns are observed. The buckling pattern can be recognized as wrinkles in the central arc segment, wrinkles in the edge arc segment, wrinkles in the web regions, or wrinkles diffused over a wide region including both the web and the edge arc segment, as shown in Figure 6.

**Figure 7:**
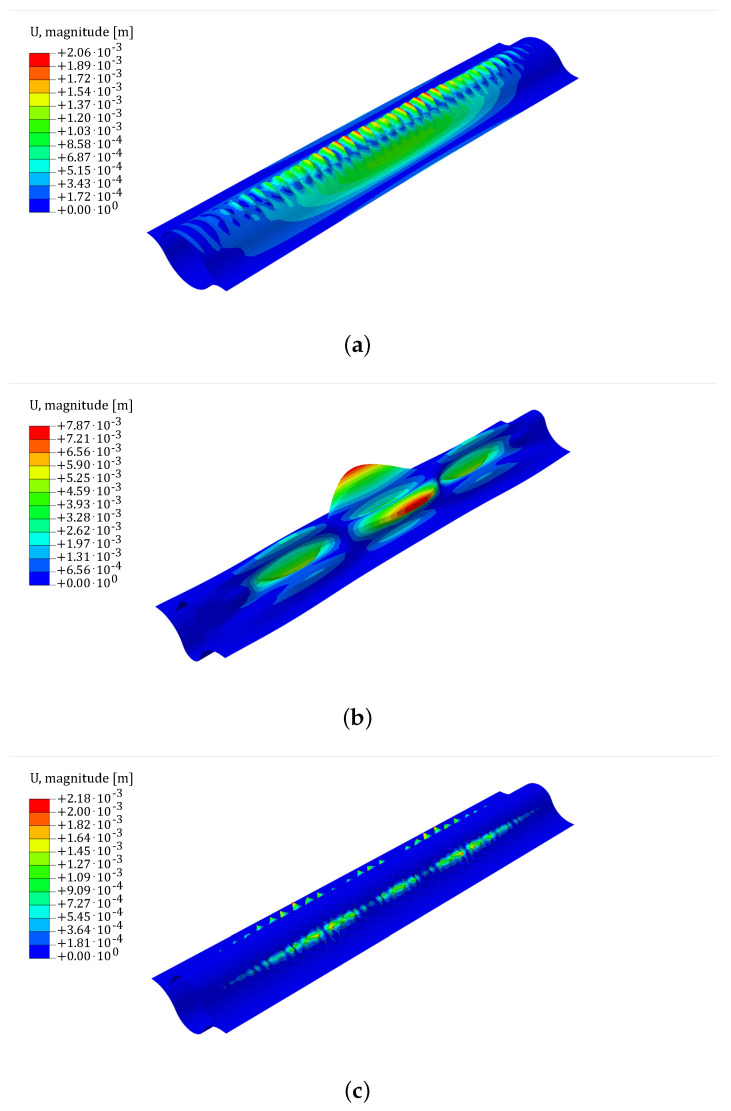
Buckling patterns for non-linear buckling in the bending moment about the *x*-axis scenario: (**a**) Wrinkles on central arc segment. (**b**) Wrinkles on edge arc segment. (**c**) Diamond wave pattern. The scale-plate is shown in the figure with the magnitude of displacements expressed in m. In the deformed configuration, the magnitude of displacements has been amplified by a factor of 5.

In the scenario of a bending moment around the *x*-axis, three non-linear buckling patterns can be distinguished: wrinkles in the central arc segment, wrinkles in the edge arc segment, and finally a diamond pattern, as shown in Figure 7.

**Figure 8:**
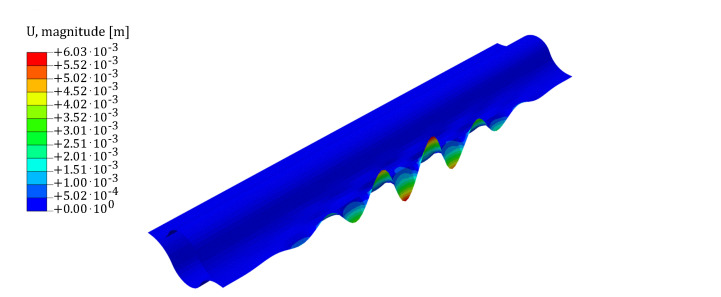
Buckling pattern for non-linear buckling in the bending moment about the *y*-axis scenario: wave pattern on compressed web. The scale-plate is shown in the figure with the magnitude of displacements expressed in m. In the deformed configuration, the magnitude of displacements has been amplified by a factor of 5.

Finally, a bending moment around the *y*-axis leads to the compression in the outer edge of the web. The only buckling pattern observed is the wrinkle pattern shown in Figure 8.

The formation of wrinkles in a structure undergoing buckling indicates that the structure is experiencing non-uniform local deformations or instabilities before reaching global collapse. These wrinkles are folds or creases that form on the surface of the structure due to local compressive stresses, and can be an early sign of potential failure or nonlinear behavior of the structure under critical buckling loads. In practical terms, the formation of wrinkles can compromise structural integrity, and may indicate that the structure is nearing its maximum load-carrying capacity. The presence of wrinkles is due to the ultra-thin nature of the CTM, which gives rise to highly localized buckling modes rather than global ones. Due to the complexity of predicting these modes analytically, surrogate models are essential for capturing this localized highly nonlinear behavior, providing a powerful tool for mitigating structural failure.

### 3.2. Surrogate Models Results

**Figure 9:**
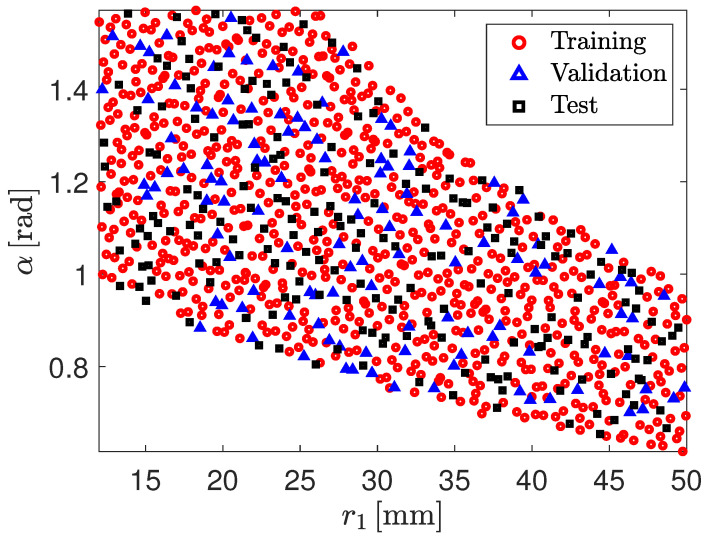
Training, validation and test datasets used to construct the NN-based surrogate models. The 680 red circles correspond to the input array of the training dataset, the 120 blue triangles correspond to the input array of the validation dataset, the 200 black squares correspond to the input array of the test dataset. (For interpretation of the references to color in this figure legend, the reader is referred to the web version of this article).

In this section, the NN-based surrogate models are presented and their predictive capability discussed. Each NN-based surrogate model is trained using data points derived FEA. Out of 1000 data points, 800 are used for training, with 680 allocated for training and 120 for validation. The remaining 200 experiments are used for testing. Those datasets are shown in Figure 9.

The result of the comparative analysis between 16 different NN architectures is presented for the NN-based surrogate model for non-linear buckling prediction in the axial loading scenario, with details given in Table 3. In Table 3, moving down the rows increases the number of layers (from 1 to 4), and moving to the right across the columns increases the total number of neurons (8-16-32-64). It can be observed that, for NNs with the same number of layers, performance improves as the number of neurons increases. With the same number of neurons, performance increases as the number of layers increases up to three. However, adding a fourth layer does not result in further performance improvement, but instead leads to a reduction in performance, especially in the case where the total number of neurons is equal to 8. Based on these considerations, we opted to employ a NN configuration with three hidden layers, having 16 neurons in the first and last hidden layers and 32 neurons in the middle hidden layer. This NN configuration has been labeled as 16-32-16. The same methodology was applied to select the NN architectures for the NN-based surrogate models for the predicting the critical bending moments around the *x* and *y* axes.

These investigations resulted in the selection of a the same NN architecture across all three surrogate models, leading to the following evaluation indices: $R^{2}=0.9987$, MAE=0.0054, and $MSE=6.7048\times 10^{-5}$, and $R^{2}=0.9628$, MAE=0.0313, and MSE=0.00178 for the *x* and *y* bending moment scenarios, respectively.

**Figure 10:**
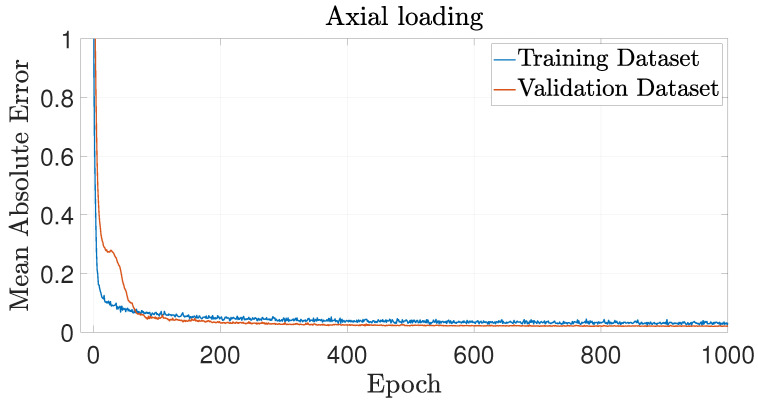
Learning curve for the NN predicting buckling behavior under axial loading, showing the MAE against the number of epochs for both the training and validation datasets.

**Figure 11:**
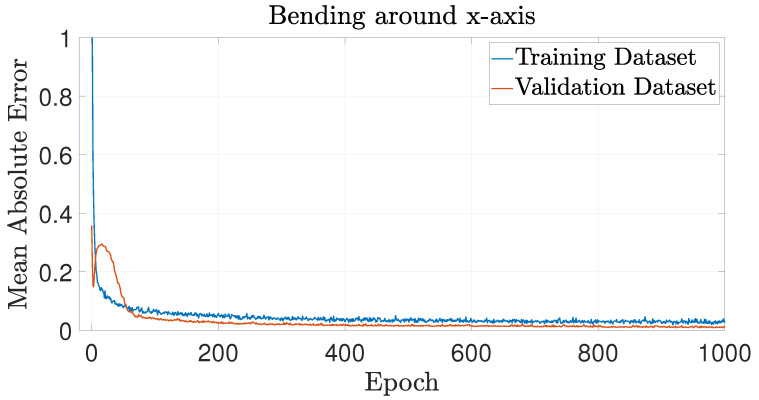
Learning curve for the NN predicting buckling behavior under pure bending around *x*-axis, showing the MAE against the number of epochs for both the training and validation datasets.

**Figure 12:**
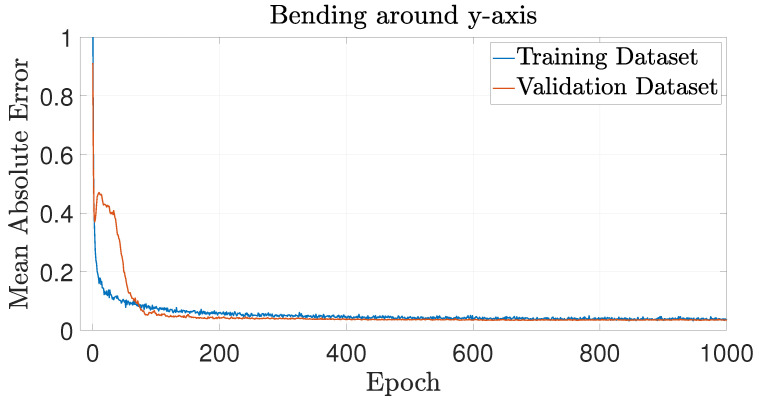
Learning curve for the NN predicting buckling behavior under pure bending around *y*-axis, showing the MAE against the number of epochs for both the training and validation datasets.

The training performance of each NN is presented in terms of MAE, evaluated on both the training and validation datasets in Figure 10, Figure 11 and Figure 12 for axial loading, bending moment around the *x*-axis and bending moment around the *y*-axis, respectively. These figures show that the MAE reaches a plateau before the end of the fixed 1000 training epochs for every NN, suggesting that the training has effectively converged.

**Figure 13:**
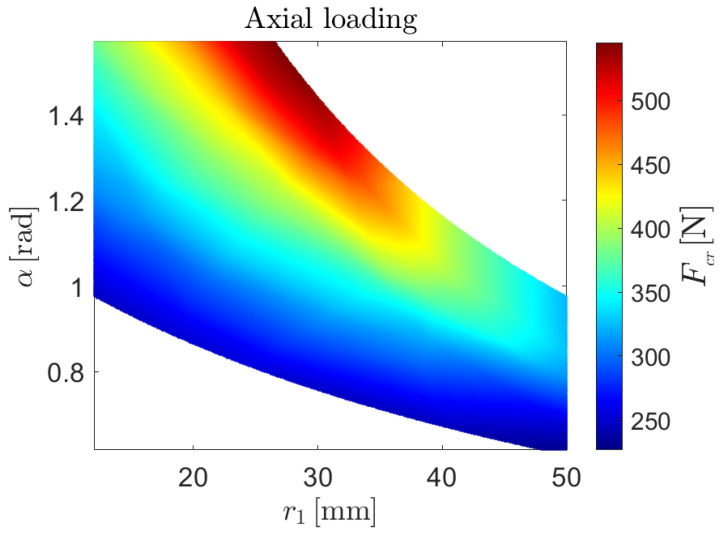
Prediction of the non-linear critical load as a function of the radius of the central arc $r_{1}$ and the subtended angle of both arc segments, α, given by the NN-based surrogate model for buckling behavior under axial loading.

**Figure 14:**
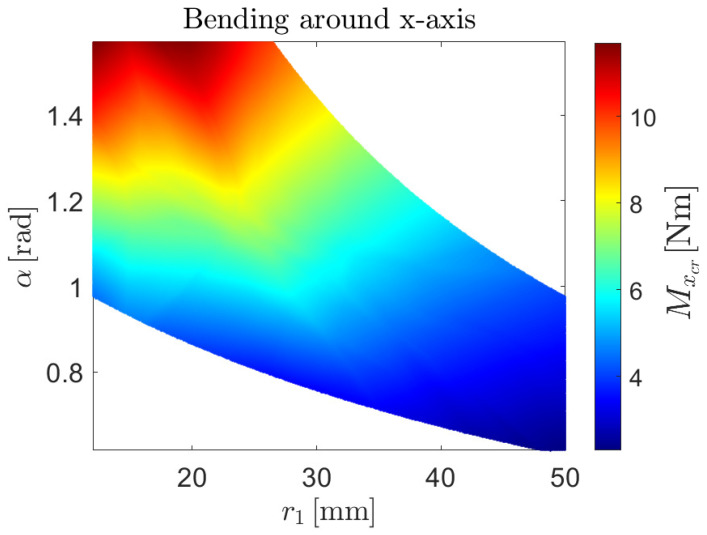
Prediction of the non-linear critical moment as a function of the radius of the central arc $r_{1}$ and the subtended angle of both arc segments, α, given by the NN-based surrogate model for buckling behavior under pure bending around the *x*-axis.

**Figure 15:**
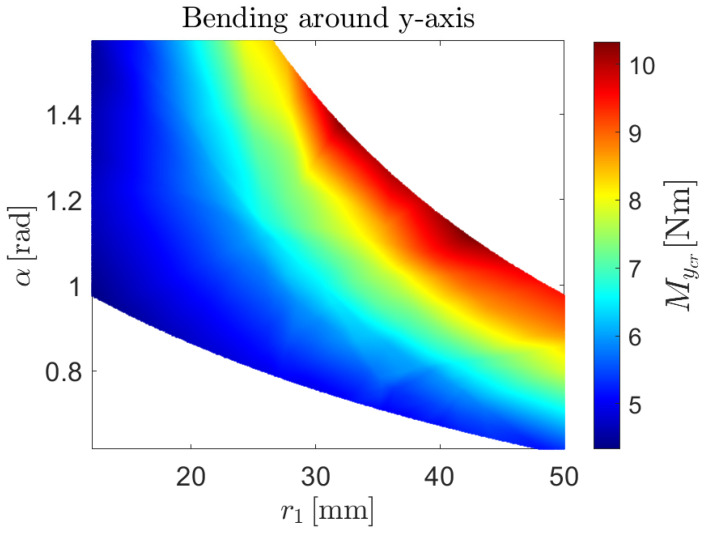
Prediction of the non-linear critical moment as a function of the radius of the central arc $r_{1}$ and the subtended angle of both arc segments, α, given by the NN-based surrogate model for buckling behavior under pure bending around the *y*-axis.

The NN-based surrogate models lead to the non-linear buckling predictions shown in Figure 13, Figure 14 and Figure 15 for the case of axial load, bending moment around the *x*-axis and bending moment around the *y*-axis, respectively.

### 3.3. Multi-Objective Optimization Results

**Figure 16:**
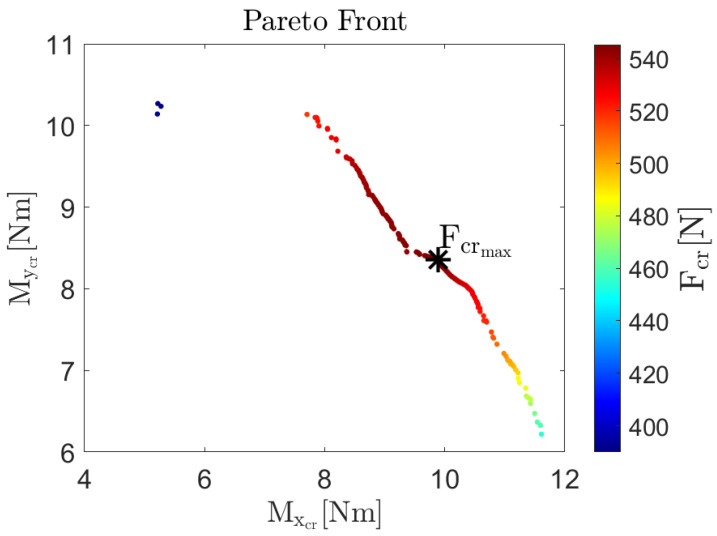
Pareto front identified using the NSGA-II algorithm with the non-linear critical buckling load under pure axial force application, the non-linear critical buckling moment under pure bending around the *x*-axis and the non-linear critical buckling moment under pure bending around the *y*-axis as objective functions and lower and upper limit for the radius of the edge arc segment $r_{2}$ as inequality constraints.

**Figure 17:**
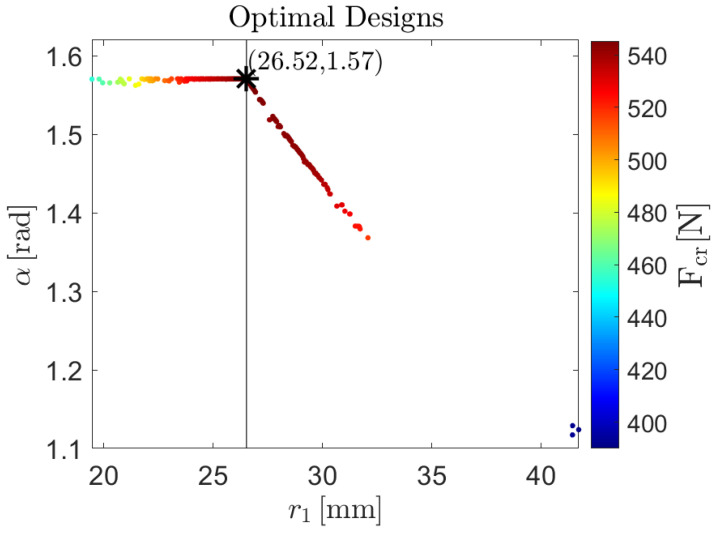
Optimal design identified using the NSGA-II algorithm with the non-linear critical buckling load under pure axial force application, the non-linear critical buckling moment under pure bending around the *x*-axis and the non-linear critical buckling moment under pure bending around the *y*-axis as objective functions and lower and upper limit for the radius of the edge arc segment $r_{2}$ as inequality constraints. The vertical line divides the optimal designs space in two regions: a left-hand region where the $r_{1}$ values range from 19.45 mm to 25.62 mm, and a right-hand region where the $r_{1}$ values range from 25.62 mm to 41.71 mm. The design point having r1=25.62 mm and α=1.57 rad lies on that vertical line.

The Pareto front and the corresponding design variables are shown in Figure 16 and Figure 17, respectively.

In Figure 16, the Pareto front is plotted in a 2D graph, with the *x*-axis showing the predicted values for the nonlinear buckling moment under pure bending around the *x*-axis, and the *y*-axis showing the predicted values for the nonlinear buckling moment under pure bending around the *y*-axis. The color represents the nonlinear buckling load under axial force application, as indicated by the color bar shown on the side of Figure 16. The maximum value for the three objective functions are: $M_{xcr}=11.71$ Nm, $M_{ycr}=10.27$ Nm, and $F_{cr}=545.26$ N. An increment of the critical buckling moment around the *x*-axis always corresponds to a decrement of the critical buckling moment around the *y*-axis; these objectives are always competing. The relations between the critical buckling moment around either the *x* or the *y* axis and the critical buckling load is not as straightforward.

In Figure 17, the cross-section parameters leading to a global optimum for buckling performance are shown. Specifically, the *x*-axis displays the value of the central arc radius, $r_{1}$, and the *y*-axis displays the value of the subtended angle of both arc segments. The minimum and maximum value for the optimal cross-sectional parameters are 19.45 mm and 41.71 mm, 1.12 rad and 1.57 rad, for $r_{1}$ and α, respectively. A vertical line divides the optimal designs space in two regions: a left-hand region and a right-hand region. The design point having $r_{1}=25.62$ mm and α=1.57 rad lies on that vertical line. In the left-hand region, the $r_{1}$ values range from 19.45 mm to 25.62 mm, while the subtended angle α takes a constant value equal to 1.57 rad. In the right-hand region, the $r_{1}$ values range from 25.62 mm to 41.71 mm, while α decreases from its uppermost value of 1.57 rad to its lowest value of 1.12 rad. In the right-hand region, the radius of the edge arc segment $r_{2}$ takes approximately a constant value equal to its lower limit of 12 mm. Once again, the points are colored according to the scale of the nonlinear buckling loads under axial force application, allowing for easy correspondence between points on the Pareto front and cross-sectional parameter pairs in the optimal design space.

## 4. Discussion

In this paper, 1000 sample designs are created, whose buckling behavior has been characterized through FEA under three different loadings: axial load, bending moment around the *x*-axis, and bending moment around the *y*-axis. One simulation, consisting of three analyses (linear buckling, non-linear static analysis, and sequential linear buckling), has been run per each loading condition over every sample design. The simulation process has been automated by developing a Python script to communicate with the Abaqus software. Worth noting is that the extent of computations in this study far exceeds that of any previous studies on buckling behavior conducted on CTMs. The sample designs and the response database thus obtained have been collected to serve as data for the machine learning process. An NN has been training for each loading scenario, and three NN-based surrogate models have been constructed. These models demonstrate good accuracy in predicting for the non-linear buckling behavior of CTMs. The surrogate models are embedded into the NSGA-II algorithm for MOO, giving a set of trad-off solutions that can be tailored to specific mission requirement.

Considering optimal solutions, the higher the $r_{1}$ value, the better the buckling performance when a bending moment around the *y*-axis is applied. Conversely, for bending moments around the *x*-axis, increasing $r_{1}$ leads to a deterioration in buckling performance. Therefore, the enhancement of the buckling performance under bending moment about perpendicular axes is always a trade-off. A further investigation of the optimal designs is conducted considering the critical buckling axial load. Specifically, two regions are identified within the space of optimal designs: a left-hand side, where the $r_{1}$ values range from 19.45 mm to 25.62 mm, and a right-hand where the $r_{1}$ values range from 25.62 mm to 41.71 mm. The critical buckling axial load increases with an increment of $r_{1}$ up to its maximum obtained in correspondence of $r_{1}=25.62$ mm, starting from which a further increment of $r_{1}$ cause the critical buckling axial load to decrease. Therefore, we distinguish a left-hand side of the optimal designs space, where $r_{1}$ is smaller than 25.62, and a right-hand side of the optimal design space, where $r_{1}$ is larger than 25.62. In the left-hand side of the optimal designs space, simultaneous increment of the non-linear buckling load and non-linear buckling moment around the *y*-axis is possible by keeping α=π/2 and increasing the value of $r_{1}$ from its lowest value of 19.45 mm up to the value of 25.62 mm. In the right-hand side of the optimal designs space, a simultaneous improvement in the non-linear buckling load and non-linear buckling moment around the *x*-axis is possible by decreasing the value of $r_{1}$ from its highest value of 41.71 mm down to 25.62 mm. Thus, it is advisable to choose the design space sides for selecting optimal solutions depending on the metric one aims to favor. It should be noted that, in the left-hand side, α is kept constant and equal to its maximum allowable value of 1.57 rad, while in the right-hand side, the α and $r_{1}$ pairs always correspond to a $r_{2}$ equal to its minim allowable value of 12 mm. This means that our engineering decision to constrain the design space has affected the Pareto front, and and alternative initial constraints on the design variables would lead to different Pareto fronts.

**Figure 18:**
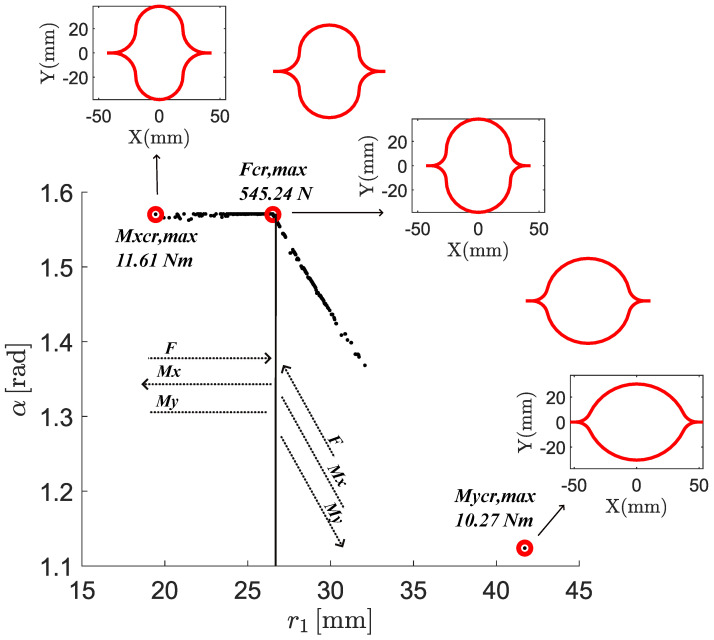
Optimal design samples with comments based on their non-linear critical loads. The dashed arrows trace the trend of nonlinear buckling loads under axial load, bending moment about the *x*-axis, and bending moment about the *y*-axis as the design variables vary. The vertical line divides the optimal designs space in two regions: a left-hand region, where the nonlinear buckling load and the non-linear buckling moment about the *y*-axis follow the same trend, and a right-hand region, where the nonlinear buckling load and the non-linear buckling moment about the *x*-axis follow the same trend. The optimal designs that maximize the nonlinear buckling moment about the *x*-axis, the nonlinear axial buckling load, and the nonlinear buckling moment about the *y*-axis are highlighted in black boxes and are shown from left to right, respectively. Intermediate configurations between these optimal designs are provided as well.

**Figure 19:**
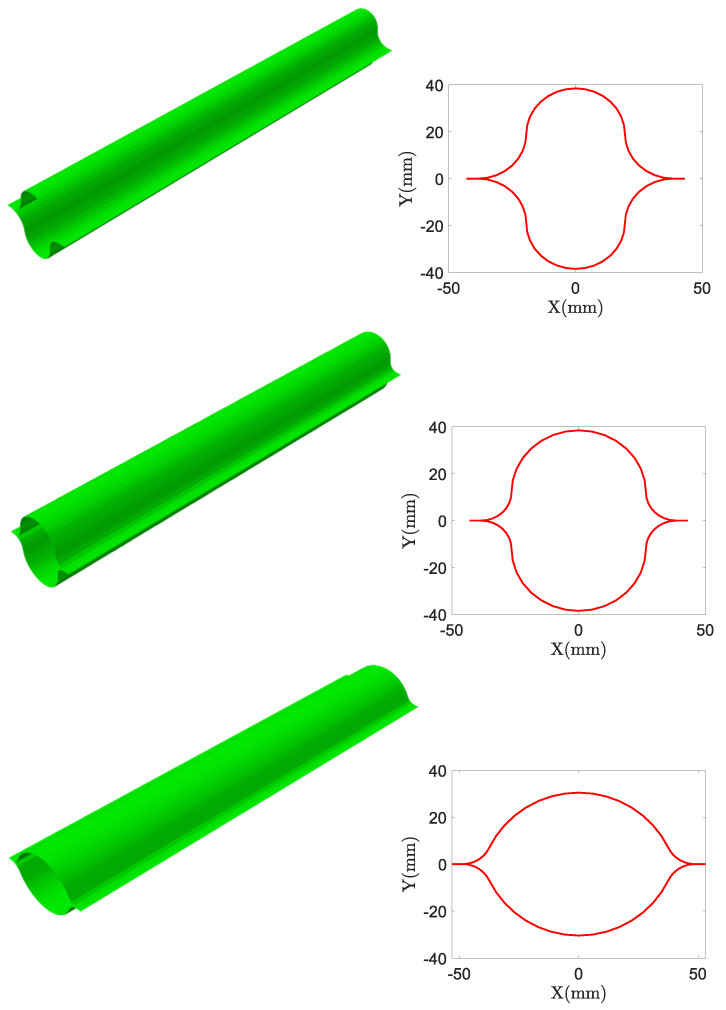
From top to bottom, the three-dimensional images show CTMs in their deployed state, along with their respective cross-sections. These images represent the designs that maximize, respectively, the nonlinear buckling moment about the *x*-axis, the nonlinear axial buckling load, and the nonlinear buckling moment about the *y*-axis.

All these findings are summarized in Figure 18. By following the orientation of the dashed arrows along the vertical line, one can trace the trend of nonlinear buckling loads under axial load, bending moment about the *x*-axis, and bending moment about the *y*-axis as the design variables vary. Three optimal configurations are highlighted in black boxes. From left to right, these configurations represent the designs that maximize the nonlinear buckling moment about the *x*-axis, the nonlinear axial buckling load, and the nonlinear buckling moment about the *y*-axis, respectively. The corresponding critical load values are $M_{xcr}=11.61\;\mathrm{Nm}$, $F_{cr}=545.24\;N$, and $M_{ycr}=10.27\;\mathrm{Nm}$. Additionally, intermediate configurations between these optimal designs are shown to provide a clearer visualization of how the cross-sections evolve within the optimal design space. Further representations of the designs that individually maximize each of the three critical loads are shown in Figure 19. In Figure 19, a three-dimensional image of these CTMs in their deployed state, along with the respective cross-section, is displayed. The order followed from left to right in Figure 18 is followed from top to bottom in Figure 19.

We recognize that optimizing the buckling behavior of CTMs under three loading conditions does not pose a high-dimensional problem, as it involves only three-objective functions and two design variables defining a relatively small design space. Consequently, the most of the decision space could have been visualized by evaluating via FEA a larger number of sample designs, without usage on surrogate models. However, even within this framework, the contribution of NN in the proposed approach resulted in a much faster design process than would have been achievable with solely time-consuming FEA. Furthermore, MOO enables design decisions informed by quantitative information rather than intuition alone, and MOO could not have been performed based solely on FEA. Instead, it necessitates models to evaluate objective functions, which can be facilitated through NN-based surrogate modeling.

In addition to providing a valuable tool for designing of CTMs with optimal buckling behavior, this work serves as an illustration of the combined NN-based surrogate models and MOO approach. We strongly believe that integrating NN with MOO is the viable solution for designing solutions within a reasonable time-frame in high-dimensional input or output spaces, with NN-based surrogate models facing the challenge of predicting complex behaviors in low time and MOO allowing us to handle multi-dimensionality. Hence, the proposed approach paves the way for increasingly complex optimizations of ultra-thin structures exhibiting highly nonlinear behaviors.

## 5. Conclusions

This paper proposes a combined NN-based surrogate model and multi-objective optimization approach to design composite lightweight CTMs with optimal buckling performance. First, we generated 1000 sample designs using the Sobol sequence method. Each design is defined by two variables: the radius of the central arc segment, $r_{1}$, and the subtended angle, α, characterizing the CTMs’ cross-section. To examine the buckling behavior of each design, we employed FEA. Specifically, we evaluated the non-linear buckling load for each design under three different loading conditions: axial load, bending moment about the *x*-axis, and bending moment about the *y*-axis. This involved performing three types of analyses (linear buckling, non-linear static analysis, and sequential linear buckling) for each design under each loading condition, totaling 3000 analyses. The computational effort in this study exceeds any prior research on the buckling behavior of CTMs. The resulting sample designs and their corresponding non-linear buckling loads for each loading scenario were compiled into a database for machine learning. Separate NNs were trained for each loading condition, using datasets divided into three parts: 680 points for training, 120 points for validation, and 200 points for testing. This approach produced three surrogate models that accurately predict the non-linear buckling behavior of CTMs, with $R^{2}$ values of 0.9906, 0.9987, and 0.9628 for axial loading, pure bending about the *x*-axis, and pure bending about the *y*-axis, respectively. These NN-based surrogate models were then incorporated into the NSGA-II algorithm for multi-objective optimization (MOO), resulting in a set of trade-off solutions that can be tailored to specific mission requirements.

In the buckling optimization framework, the proposed approach resulted in a faster design process than would have been achievable with solely time-consuming FEA. In fact, NN-based surrogate models predict the buckling behavior for newer sample designs much faster than FEA, with the prediction time reduced to a fraction of a second, compared to the several minutes required by FEA. Furthermore, the application of multi-objective optimization enabled the identification of trends that would have been challenging to detect through intuition alone. By utilizing multi-objective optimization based on quantitative data derived from surrogate models, we were able to elucidate how improvements in buckling performance under axial loading correlate with enhancements in buckling performance under bending moments about the *y*-axis, but only within the design space where the cross-sectional radius of curvature is less than 26.52 mm. For larger radii of curvature, improvements in axial load buckling performance are instead associated with improvements in buckling performance under bending moments about the *x*-axis. Consequently, we advise designers to select design spaces tailored to the anticipated loading conditions of the boom during operational scenarios. These findings were achievable solely due to the capacity of MOO to manage multiple objective functions concurrently. The efficacy of this MOO was contingent upon the availability of surrogate models for buckling behavior. Without such surrogate models, it would not have been feasible to deviate from intuitive assumptions and to guide the CTM design based on rigorous quantitative analysis.

In addition to serving as a valuable tool for designing CTMs with optimal buckling behavior, the case study acts as a benchmark to illustrate the advantages of integrating NN and MOO in the design process of a broader range of lightweight composite structures. This integration facilitates the discovery of feasible solutions within a reasonable time-frame, leveraging the reduced computational costs of NN evaluations compared to FEA. This enhanced computational efficiency supports a shift from intuition-based design decisions to data-driven approaches. By tackling the challenge of multidimensionality in the decision-making process, a data-driven approach reveals crucial solutions that intuition alone might overlook. We firmly believe that integrating NN and MOO represents the path towards innovative designs for lightweight composite structures.

## Figures and Tables

**Table 1:** Design variables with lower and upper bounds.

Design Variable	Lower Bound	Upper Bound
$r_{1}$	12 mm	50 mm
α	π/5 rad	π/2 rad

**Table 2:** Hyper-parameters for the NSGA-II algorithm.

Hyper-Parameter	
No. of populations	1000
No. of offspring	100
Sampling operator	latin hypercube sampling (LHS)
Selector operator	binary tournament
Cross-over operator	simulated binary crossover (SBX)
$\eta_{c}$ (SBX)	15
Mutation operator	polynomial mutation (PM)
$\eta_{c}$ (PM)	20
Mutation rate	0.9
Max generation	100
Eliminate Duplicate	Active

**Table 3:** Evaluation metrics $R^{2}$, MSE, MAE of the NN-based surrogate model for non-linear buckling prediction in the axial loading scenario, obtained with 16 different NN architectures. Moving down the rows increases the number of layers (from 1 to 4), and moving to the right across the columns increases the total number of neurons (8-16-32-64).

	8 Neurons	16 Neurons	32 Neurons	64 Neurons
**1 layer**	**8**	**16**	**32**	**64**
$R^{2}$	0.9744	0.9840	0.9864	0.9870
MSE	0.00106	0.00066	0.00056	0.00053
MAE	0.0259	0.0204	0.0192	0.0185
**2 layers**	**4-4**	**8-8**	**16-16**	**32-32**
$R^{2}$	0.9820	0.9844	0.9878	0.9894
MSE	0.00074	0.00064	0.00050	0.00043
MAE	0.0217	0.0192	0.0175	0.0153
**3 layers**	**2-4-2**	**4-8-4**	**8-16-8**	**16-32-16**
$R^{2}$	0.9848	0.9867	0.9890	0.9906
MSE	0.00062	0.00055	0.00045	0.00039
MAE	0.0195	0.0174	0.0157	0.0144
**4 layers**	**2-2-2-2**	**4-4-4-4**	**8-8-8-8**	**16-16-16-16**
$R^{2}$	0.9683	0.9862	0.9877	0.9891
MSE	0.00130	0.00057	0.00050	0.00045
MAE	0.028	0.0179	0.0168	0.0154

## Data Availability

Data will be made available on request.
